# Changes in plasma and erythrocyte omega-6 and omega-3 fatty acids in response to intravenous supply of omega-3 fatty acids in patients with hepatic colorectal metastases

**DOI:** 10.1186/1476-511X-12-64

**Published:** 2013-05-07

**Authors:** Omer Al-Taan, James A Stephenson, Laura Spencer, Cristina Pollard, Annette L West, Philip C Calder, Matthew Metcalfe, Ashley R Dennison

**Affiliations:** 1 Department of Hepatobiliary and Pancreatic Surgery, University Hospitals of Leicester NHS Trust, Gwendolen Road, Leicester LE5 4PW, United Kingdom; 2 Human Development and Health Academic Unit, Faculty of Medicine, University of Southampton, Southampton General Hospital, Tremona Road, Southampton SO16 6YD, United Kingdom; 3 National Institute for Health Research Southampton Biomedical Research Centre, University of Southampton and University Hospital Southampton NHS Foundation Trust, Tremona Road, Southampton SO16 6YD, United Kingdom

**Keywords:** Parenteral nutrition, Fish oil, Omega-3 fatty acids, Eicosapentaenoic acid, Docosahexaenoic acid, Arachidonic acid, Liver metastases

## Abstract

**Background:**

Eicosapentaenoic acid (EPA) and docosahexaenoic acid (DHA) are functionally the most important omega-3 polyunsaturated fatty acids (PUFAs). Oral supply of these fatty acids increases their levels in plasma and cell membranes, often at the expense of the omega-6 PUFAs arachidonic acid (ARA) and linoleic acid. This results in an altered pattern of lipid mediator production to one which is less pro-inflammatory. We investigated whether short term intravenous supply of omega-3 PUFAs could change the levels of EPA, DHA, ARA and linoleic acid in plasma and erythrocytes in patients with hepatic colorectal metastases.

**Methods:**

Twenty patients were randomised to receive a 72 hour infusion of total parenteral nutrition with (treatment group) or without (control group) omega-3 PUFAs. EPA, DHA, ARA and linoleic acid were measured in plasma phosphatidylcholine (PC) and erythrocytes at several times points up to the end of infusion and 5 to 12 days (mean 9 days) after stopping the infusion.

**Results:**

The treatment group showed increases in plasma PC EPA and DHA and erythrocyte EPA and decreases in plasma PC and erythrocyte linoleic acid, with effects most evident late in the infusion period. Plasma PC and erythrocyte EPA and linoleic acid all returned to baseline levels after the 5–12 day washout. Plasma PC DHA remained elevated above baseline after washout.

**Conclusions:**

Intravenous supply of omega-3 PUFAs results in a rapid increase of EPA and DHA in plasma PC and of EPA in erythrocytes. These findings suggest that infusion of omega-3 PUFAs could be used to induce a rapid effect especially in targeting inflammation.

**Trial registration:**

http://www.clinicaltrials.gov identifier NCT00942292

## Background

Polyunsaturated fatty acids (PUFAs) have important roles in membrane structure and function, cell signalling and regulation of gene expression, and as substrates for synthesis of lipid mediators involved in inflammation, immunity, coagulation, smooth muscle contraction and many other physiological responses [1]. The two main families of PUFAs are the omega-6 and omega-3 families. The main dietary omega-6 PUFA is linoleic acid [2] and its derivative, arachidonic acid (ARA), is important with regard to all of the roles mentioned above [2]. The main dietary omega-3 PUFA is α-linolenic acid [2]. Its derivatives, eicosapentaenoic acid (EPA) and docosahexaenoic acid (DHA), are again involved in the roles mentioned above [3-5], although it is important to note that conversion of α-linolenic acid to EPA, and especially to DHA, appears to be limited in humans [6]. Omega-6 and omega-3 PUFAs frequently compete with one another for metabolism and often act in an opposing manner to one another [2-5]. This is especially so for ARA and EPA with regard to inflammatory processes [7-10]. Hence achieving the correct “balance” between specific members of the omega-6 and omega-3 PUFA families is likely to be important for good health and for improved patient outcomes. Conversely a disturbed balance may be associated with poorer health and poorer patient outcome. Because of the high dietary abundance of omega-6 PUFAs [11] and the relatively poor abundance of omega-3 PUFAs, omega-6 fatty acids predominate over omega-3 in blood lipids, blood cells and most tissues [2-4]. However, increased oral intake of EPA and DHA results in an increase in their abundance in blood lipids [12-15], in blood cells [12-15], and in tissues [16-18]. Likewise, intravenous supply of EPA and DHA increases the content of those fatty acids in blood lipids [19-22] and in blood cells [23,24].

Patients who undergo major surgery have a risk of infectious complications and these may increase morbidity, length of hospital stay and mortality. It is possible that the risk of infection, of further complications and of being unable to recover from the complications is increased by a high status of omega-6 fatty acids and/or a low status of omega-3 fatty acids, especially EPA and DHA [7,25]. Therefore, providing EPA and DHA in advance of a surgical insult might reduce the risk of adverse consequences [25]. Intravenous administration of EPA and DHA, in the form of a lipid emulsion containing some fish oil, is a strategy to easily and rapidly increase omega-3 PUFA supply and status [26-29]. The impact of intravenous fish oil has been explored in some surgical settings, mainly related to colorectal cancer resection [25], but not in the context of removal of liver metastases. The aim of the present study was to examine the fatty acid composition of plasma phosphatidylcholine (PC), the major phospholipid in the circulation, and erythrocytes during and after short term intravenous administration of a lipid emulsion that includes fish oil in patients awaiting surgery for removal of liver colorectal metastases.

## Methods

### Study design

This study was part of a double blind randomised controlled trial examining the effect of fish oil on human colorectal metastases. Ethical approval was obtained from the Leicestershire, Northamptonshire and Rutland Research Ethics Committee (REC number 06/Q2501/16) and approval for the study was obtained from the Medicines and Healthcare Products Regulatory Agency (EudraCT number: 2006-000044-71). The trial is registered at http://www.clinicaltrials.gov with identifier NCT00942292. Patients gave written informed consent.

### Patient selection and lipid administration

Patients who had hepatic colorectal metastases larger than 3 cm in diameter were identified from the University Hospitals of Leicester Hepatobiliary Cancer Multi-Disciplinary Team meetings. Patients where the lesion was deemed amenable for curative resection were invited to enrol in the study. To be eligible for inclusion, patients had to have normal liver function tests and normal plasma lipid concentrations. Twenty patients were enrolled in the trial. Patients were aged 44 to 80 years and there were 9 men and 11 women. Patients received total parenteral nutrition for 72 hr continuously at a rate of 1.5 ml per kilogram body weight per hr via a peripherally inserted central catheter and only drank water by mouth. The parenteral nutrition was in the form of 2000 ml Nutriflex basal® (B Braun, Melsungen, Germany) compounded with 500 ml Lipidem® 20% (B Braun) in the treatment group or with 500 ml Lipofundin® MCT 20% (B Braun) in the control group. Lipidem® (also known as Lipoplus®) is a 50:40:10 (vol/vol/vol) mix of medium-chain triglycerides, soybean oil and fish oil; each 100 ml of Lipidem® will typically contain about 1.25 g EPA plus DHA (0.74 g EPA and 0.51 g DHA). Lipofundin® MCT is a 50:50 (vol/vol) mix of medium-chain triglycerides and soybean oil. Median age (69 years in the control group and 63 years in the treatment groups) and the balance of men and women (5:6 in the control group and 4:5 in the treatment group) did not differ between the two groups.

### Blood sampling and processing

Blood samples were taken immediately before the infusion and 1, 3, 6, 20, 44, 68 and 72 hr after starting the infusion. On finishing the infusion, patients were discharged home and re-admitted within 2 weeks (5 to 12 days; mean 9 days) to have resection of the liver lesion; at this stage one further blood sample was taken. Blood was collected into heparin as anti-coagulant. Blood was centrifuged to obtain plasma and an erythrocyte pellet. These were separated and stored at −80°C until analysis.

### Fatty acid composition of plasma phosphatidylcholine and erythrocytes

Total lipid was extracted from plasma or erythrocytes using chloroform/methanol (2:1 vol/vol). PC was separated from other plasma lipids by solid phase extraction on Bond Elut cartridges (Varian, Palo Alto, CA, USA). Plasma PC and erythrocyte lipids were saponified and fatty acid methyl esters formed by heating at 50°C for 2 hr in the presence of sulphuric acid containing 2% methanol. Fatty acid methyl esters were extracted into hexane and concentrated by evaporation under nitrogen. Fatty acid methyl esters were separated by gas chromatography on a Hewlett Packard 6890 gas chromatograph (Hewlett Packard, CA, USA) fitted with a BPX70 column (SGE Europe, Milton Keynes, Bucks, UK). Running conditions were as described elsewhere [15]. Fatty acids were identified by comparison of retention times with those of authentic standards. Data are expressed as percentage contribution to the total fatty acid pool. The main focus of the data shown in this paper is on EPA, DHA, ARA and linoleic acid.

### Statistical analysis

Data are shown as mean + SEM. Data were first analysed using two-factor ANOVA (factors: time and group) for repeated measures. This was followed by analysis of effects within a group and by pairwise comparisons between groups as appropriate. Statistical analysis was performed using GraphPad Prism for windows (GraphPad Software Inc., CA, USA). Differences were considered significant at P < 0.05.

## Results

The control group (n = 11) received total parenteral nutrition for 72 hr with the lipid component being in the form of a 50:50 (vol/vol) mixture of medium-chain triglycerides and soybean oil. The treatment group (n = 9) received a 50:40:10 (vol/vol/vol) mixture of medium-chain triglycerides, soybean oil and fish oil. Blood samples were taken immediately before infusion and 1, 3, 6, 20, 44, 68 and 72 hr after starting the infusion. A further sample was collected 5 to 12 days (mean 9 days) after the end of the infusion period. The fatty acid compositions of plasma PC and erythrocytes were determined. These did not differ between groups at baseline (i.e. prior to infusion).

### Plasma phosphatidylcholine fatty acids

**Figure 1:**
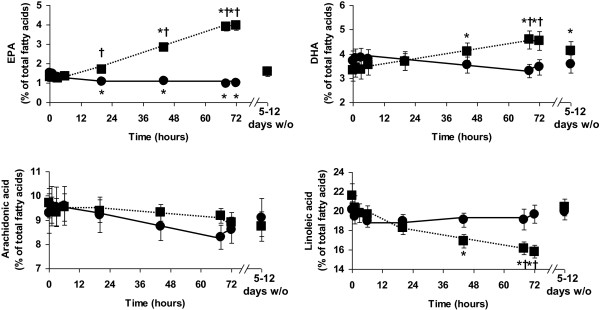
**Plasma PC eicosapentaenoic acid (EPA), docosahexaenoic acid (DHA), arachidonic acid and linoleic acid before (baseline), during (1 hr to 72 hr) and 5–12 days after stopping infusion of a mixture of medium-chain triglycerides and soybean oil (control: filled circles joined by solid lines) or medium-chain triglycerides, soybean oil and fish oil (fish oil: filled squares joined by dotted lines).** Data are mean ± SEM. *indicates significantly different from baseline value in the same group. ^†^indicates significantly different from control group at the same timepoint. 5–12 days w/o indicates the samples collected 5 to 12 days after stopping the infusions.

There was a significant effect of time (P < 0.001) and group (P < 0.001) and a significant time x group interaction (P < 0.001) for plasma PC EPA. Plasma PC EPA showed a small, but significant, decrease in the control group (P = 0.004), with the decrease apparent after 20 (P = 0.010 vs baseline), 44 (P = 0.019), 68 (P = 0.002) and 72 (P = 0.003) hr of infusion (Figure 1). Plasma PC EPA showed a marked and significant increase in the treatment group (P < 0.001), with the increase apparent after 20 (P = 0.066 vs baseline), 44 (P < 0.001), 68 (P < 0.001) and 72 (P < 0.001) hr of infusion (Figure 1). Plasma PC EPA was higher in the treatment group than in the control group at 20, 44, 68 and 72 hr (all P < 0.001). Plasma PC EPA returned to baseline values in both control and treatment groups after the 5 to 12 day washout period (Figure 1).

There was a significant effect of time (P < 0.001) and a significant time x group interaction (P < 0.001) for plasma PC DHA. Plasma PC DHA did not change significantly in the control group (P > 0.05; Figure 1). Plasma PC DHA increased in the treatment group (P < 0.001), with the increase apparent at 44 (P = 0.031 vs baseline), 68 (P = 0.008) and 72 (P = 0.011) hr (Figure 1). Plasma PC DHA was higher in the treatment group than in the control group at 68 (P = 0.007) and 72 (P = 0.067) hr. Plasma PC DHA remained elevated above baseline levels (P = 0.050) in the treatment group after the 5 to 12 day washout period (Figure 1).

There was a significant effect of time (P < 0.001) and a strong trend toward a significant time x group interaction (P = 0.062) for plasma PC ARA. Plasma PC ARA tended to decrease in the both groups (Figure 1). However, there were no significant differences from baseline in either group and there were no differences between groups at any timepoint.

There was a significant effect of time (P < 0.001) and a significant time x group interaction (P < 0.001) for plasma PC linoleic acid. Plasma PC linoleic acid did not change in the control group (P > 0.05; Figure 1). Plasma PC linoleic acid decreased in the treatment group (P < 0.001). In the treatment group plasma PC linoleic acid was lower than baseline at 44 (P = 0.019), 68 (P = 0.008) and 72 (P = 0.004) hr. Plasma PC linoleic acid differed between the control and treatment groups at 68 (P = 0.038) and 72 (P = 0.009) hr. Plasma PC linoleic acid returned to baseline values after the 5 to 12 day washout period (Figure 1).

As a result of these changes in EPA, DHA and linoleic acid content of plasma PC the omega-6 to omega-3 ratio was significantly decreased late in the infusion period in the treatment group (at 20, 44, 68 and 72 hr). There was no difference in the proportion of any other fatty acid in plasma PC between the two groups or over time.

### Erythrocyte fatty acids

**Figure 2:**
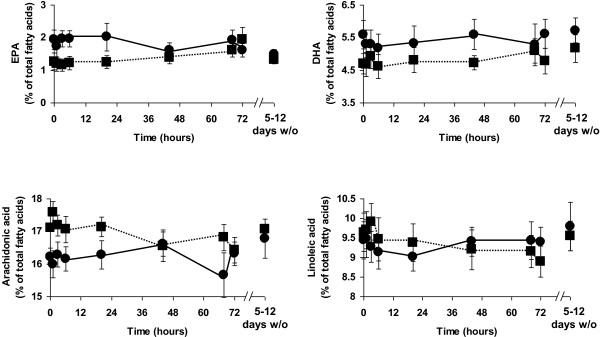
**Erythrocyte eicosapentaenoic acid (EPA), docosahexaenoic acid (DHA), arachidonic acid and linoleic acid before (baseline), during (1 hr to 72 hr) and 5–12 days after stopping infusion of a mixture of medium-chain triglycerides and soybean oil (control: filled circles joined by solid lines) or medium-chain triglycerides, soybean oil and fish oil (fish oil: filled squares joined by dotted lines).** Data are mean ± SEM. There were no significant pairwise differences between timepoints within a group or between groups at any timepoint. 5–12 days w/o indicates the samples collected 5 to 12 days after stopping the infusions.

There was a strong trend toward a significant time x group interaction for erythrocyte EPA (P = 0.054). Erythrocyte EPA did not change significantly in the control group (Figure 2), but increased in the treatment group late in the infusion period (P < 0.005) (Figure 2). However, further analysis did not show any significant differences compared to baseline erythrocyte EPA or compared with the control group. Erythrocyte EPA returned to baseline values in the treatment group after the 5 to 12 day washout period (Figure 2). Erythrocyte DHA and ARA did not change significantly in either group and the groups did not differ at any timepoint (Figure 2). There was a significant effect of time (P = 0.011) and a significant time x group interaction (P = 0.012) for erythrocyte linoleic acid. Erythrocyte linoleic acid did not change in the control group but decreased in the treatment group late in the infusion period before returning to baseline levels at the 5 to 12 day washout (Figure 2). However, further analysis did not show any significant differences compared to baseline erythrocyte linoleic acid or compared with the control group. There was no difference in the proportion of any other fatty acid in erythrocytes between the two groups or over time.

## Discussion

This current study shows that intravenous infusion of omega-3 PUFAs in the form of Lipidem® (B. Braun, Melsungen, Germany) induces a fairly rapid and marked increase in EPA and DHA in plasma PC and a small increase of EPA in erythrocytes. Amongst these changes, the elevation of EPA in plasma PC occurred the earliest. Interestingly in the control group not receiving fish oil-type omega-3 PUFAs there was a small decline in plasma PC EPA. The effect of the infusion of omega-3 PUFAs on plasma PC and erythrocyte EPA was reversed when the infusion was stopped. In contrast, elevated DHA was retained in plasma PC beyond the end of the infusion period. The retention of DHA in plasma PC was more marked in some individuals that others. Overall these data indicate that infused omega-3 PUFAs, especially EPA, are rapidly incorporated into plasma PC and also into erythrocytes and that turnover of EPA and DHA in plasma PC is different so that there is retention of DHA after supply is terminated even when EPA has returned to its starting level. Thus, this study provides further evidence that EPA and DHA may be handled differently in the body. The preferential retention of DHA has been demonstrated in plasma phospholipids [13], platelets [30], white blood cells [13] and erythrocytes [12,31] after oral dosing of omega-3 PUFAs. These observations might indicate a special functional significance or importance of DHA over EPA

The rapid appearance of EPA and DHA with intravenous infusion is an advantage over oral supply where appearance of these fatty acids is slower [12-15,32]. This is most likely because oral intake of omega-3 PUFAs must be followed by the processes of digestion and absorption before the fatty acids can be incorporated into plasma lipids and of further processing before they can be incorporated into cellular lipids. These processes all take time and depend on other factors such as the fat content and macronutrient composition of the meal. In contrast, intravenous infusion provides the fatty acids directly into the bloodstream introducing them into blood lipids, like PC, and directly exposing cells very quickly. A second aspect of intravenous infusion that will favour incorporation of EPA and DHA is the dose that can be administered. Studies of oral dosing typically use 1 to 4 g EPA + DHA per day, although higher doses have been used in some studies. In contrast, intravenous administration can easily provide more than 10 g of EPA + DHA on a daily basis. The higher dose will promote quicker incorporation and also a higher level of incorporation than is possible with oral supply. It is important to note that this high level of intravenous omega-3 PUFAs was well tolerated in all patients and there were no adverse reactions shown by study participants. Again this is an advantage over oral supply where moderate to high doses of fish oil can be associated with adverse gastrointestinal reactions.

The increase in EPA and DHA, which was mirrored by a decrease in the omega-6 fatty acid linoleic acid, resulting in decrease in the omega-6 to omega-3 PUFA ratio could be functionally important especially with regard to inflammation [7-10] and perhaps also immune function [33] and blood coagulation [34]. The infusion of EPA + DHA promotes an anti-inflammatory and anti-coagulatory environment that would be an advantage in many patient groups including acute severe pancreatitis, sepsis, head trauma and even in advance of major gastrointestinal or hepatic surgery. In the current study the functional implications of the fatty acid changes described were not investigated.

Erythrocytes from patients receiving intravenous fish oil did not show an elevation of DHA, despite the small elevation of EPA. This suggests a slower rate of incorporation of DHA than EPA into erythrocytes and that the period of infusion (72 hr) was insufficient for net DHA incorporation to occur. Browning et al. [15] demonstrated slower appearance of DHA than EPA into erythrocytes of healthy subjects consuming oral supplements containing EPA and DHA. Once again these observations indicate a slower turnover of DHA than EPA in cells.

The control group received a mixture of soybean oil and medium-chain triglycerides. This mix is fairly rich in linoleic acid, although less so than traditional soybean oil lipid emulsions [35]. One interesting observation is that plasma PC EPA decreased slightly but significantly in the control group. Given the role of EPA in limiting inflammation and preventing coagulation, the decline seen in the control group suggests an undesirable effect of infusing lipid that does not contain preformed EPA.

In the clinical setting, the overall aim of the infusion protocol used here would be to enrich cells and tissues with biologically active omega-3 PUFAs in order to slow or reduce tumour growth and to promote a favourable response to a subsequent insult such surgery. Such a favourable response might involve prevention of excessive inflammation and reducing the likelihood of immune paralysis. Here, erythrocyte fatty acids were measured as a surrogate for those in tissue. At the end of the infusion period EPA had increased in erythrocytes of those patients receiving the intravenous fish oil. However, the infusion period was stopped some days before surgery and this resulted in a reversal of the infusion-induced fatty acid composition change in erythrocytes. This may also have occurred in tissues including the liver. It will be important in future studies to prolong the infusion period up to the time of surgery, in order to maximise the likelihood of establishing a beneficial impact of omega-3 fatty acids on the response to surgery, and to sample liver tissue in order to confirm that its fatty acid composition is modified. Furthermore, it will be important to link changes in plasma, blood cell and tissue fatty acid composition to biological effects such as the concentrations of lipid mediators and cytokines and to clinical outcomes.

## Conclusions

Intravenous supply of omega-3 PUFAs results in a rapid increase of EPA and DHA in plasma PC and of EPA in erythrocytes, suggesting that infusion of omega-3 PUFAs could be used to induce a rapid effect especially in targeting inflammation.

## Abbreviations

ARA: Arachidonic acid; DHA: Docosahexaenoic acid; EPA: Eicosapentaenoic acid; PC: Phosphatidylcholine; PUFA: Polyunsaturated fatty acid.

## Competing interests

ARD, MSM, JS, OAT and CP have received educational travelling grants from B. Braun. PCC has received speaking fees from B. Braun, Fresenius Kabi, Baxter Healthcare, Abbott Nutrition, and Nestle and has received research funding from B. Braun and Abbott Nutrition.

## Authors’ contributions

MM and ARD designed the study, obtained ethical approval and identified suitable patients for recruitment. OAT, JAS, LS and CP recruited the patients. OAT, JAS and LS supervised the infusions, collected blood samples and isolated the plasma and erythrocytes. OAT, JS, and ALW performed the gas chromatography under the supervision of PCC. OAT, JS and PCC analysed the data and wrote the paper. All authors read and approved the paper.
